# Human-Assisted Invasions of Pacific Islands by *Litoria* Frogs: A Case Study of the Bleating Tree Frog on Lord Howe Island

**DOI:** 10.1371/journal.pone.0126287

**Published:** 2015-05-11

**Authors:** T. Lynette Plenderleith, Katie L. Smith, Stephen C. Donnellan, Richard D. Reina, David G. Chapple

**Affiliations:** 1 School of Biological Sciences, Monash University, Clayton, Victoria, Australia; 2 Museum Victoria, Division of Sciences, Melbourne, Victoria, Australia; 3 South Australian Museum, Adelaide, South Australia, Australia; Department of Agriculture, AUSTRALIA

## Abstract

There are substantial differences among taxonomic groups in their capacity to reach remote oceanic islands via long-distance overwater dispersal from mainland regions. Due to their permeable skin and intolerance of saltwater, amphibians generally require human-assisted dispersal to reach oceanic islands. Several *Litoria* frog species have been introduced to remote islands throughout the Pacific Ocean region. Lord Howe Island (LHI) is an oceanic island that lies approximately 600 km east of the Australian mainland and has a diverse, endemic biota. The bleating tree frog (*Litoria dentata*) is native to mainland eastern Australia, but was accidentally introduced to LHI in the 1990s, yet its ecology and potential impact on LHI has remained unstudied. We used a mitochondrial phylogeographical approach to determine that *L*. *dentata* was introduced from the Ballina region in northeastern New South Wales. The founding population was likely accidentally introduced with cargo shipped from the mainland. We also completed the first detailed investigation of the distribution, ecology and habitat use of *L*. *dentata* on LHI. The species is widespread on LHI and is prevalent in human habitat, cattle pasture and undisturbed forest. We discuss the potential impact of introduced *Litoria* species on Pacific islands and outline what biosecurity protocols could be implemented to prevent the introduction of further amphibian species to the ecologically sensitive oceanic area.

## Introduction

Large areas of open water, particularly seawater, present formidable barriers to dispersal and colonisation for most terrestrial species. Remote oceanic islands frequently lack faunal components that are present in the neighbouring mainland regions [1, 2]. They are often depauperate in mammals [3] and amphibians [4], having been colonised by taxa more able to traverse oceans, such as invertebrates [5], squamate reptiles [6], and birds [7]. Yet, oceanic island ecosystems are particularly vulnerable to invasion by non-native species [8–10], due to a high abundance of island endemics that are susceptible to invaders due to smaller population sizes, specific life history traits, and lack of appropriate defences [11]. Offshore islands present opportunities for colonisation by non-native species by offering suitable habitat and reduced pressures (such as predation, competition and parasites) from other organisms that are found on the mainland.

The Pacific is the largest ocean in the world, yet several frog species have managed to colonise oceanic islands in the region through accidental and deliberate human-assisted dispersal [12,13]. Cane toads are arguably the most infamous introduced amphibian in the region, having been introduced in attempts to control invertebrate pests (reviewed in Kraus 2009 [13]). Similarly, the coqui frog from Puerto Rico has invaded Hawai’i after being introduced through the nursery trade [14–16]. However, here we focus on the Australian frog genus *Litoria*, which contains several species that have been introduced to Pacific islands ([12,13], S1 Table).

**Fig 1:**
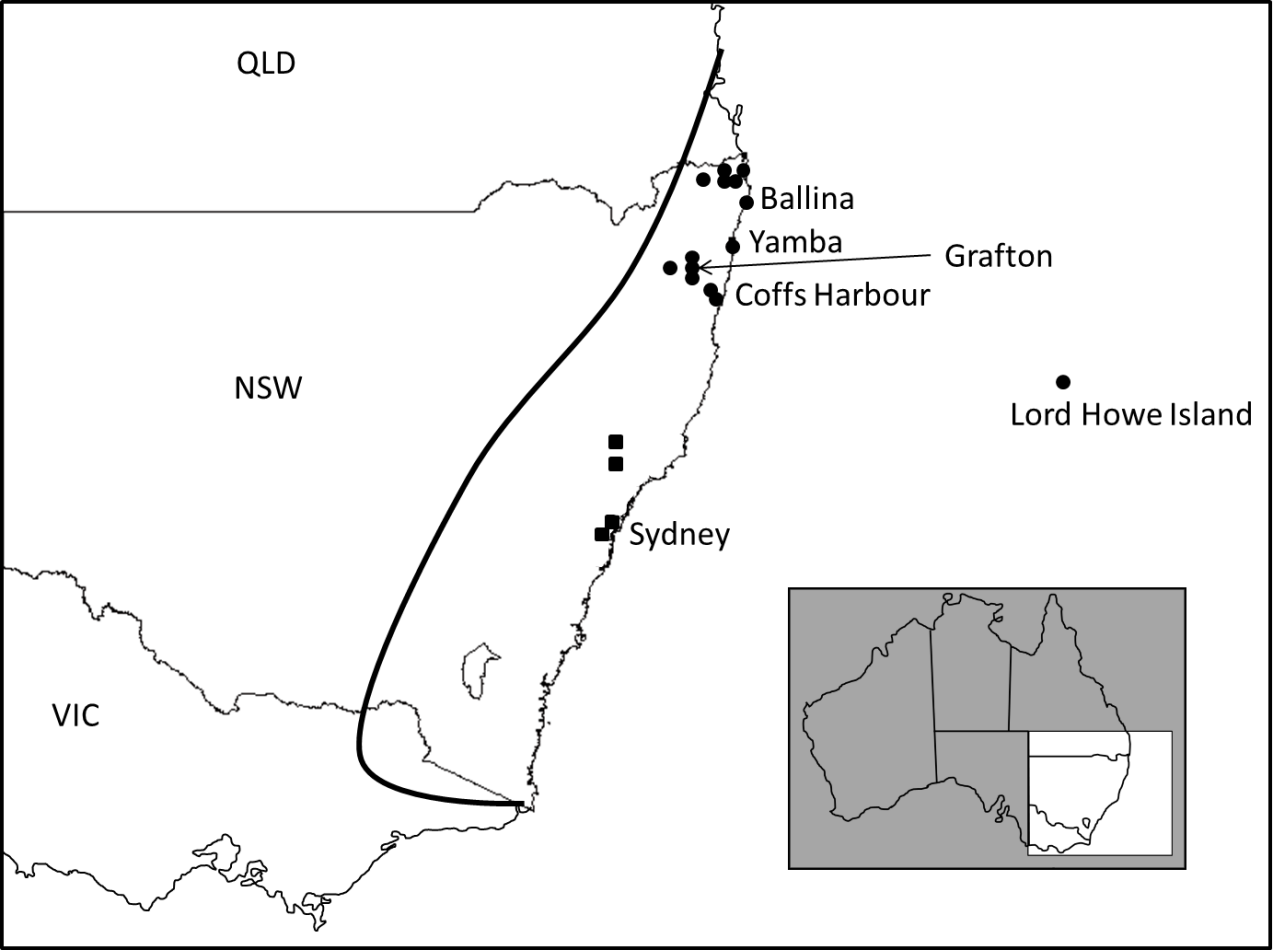
Map showing the collection localities in eastern Australia of *Litoria dentata* samples. The distribution of the northern lineage is indicated with solid black circles and the distribution of the southern lineage highlighted with solid black squares. The approximate native range of *L*. *dentata* is indicated with a solid black line. Inset shows location of map within Australia.

Many *Litoria* species have been documented to be successful invaders of Pacific islands [17,18]. The impact of these species seems to be restricted mostly to predation pressure on native species (although the suspected introduction of disease to New Zealand frogs by *Litoria raniformis*, is also important [19]), but as yet the invasion ecology of the genus has not been studied extensively (as reviewed in Lever 2003 [12]). Here we focus on the accidental introduction of the bleating tree frog (*Litoria dentata*) to Lord Howe Island (LHI) [12,13,20]. *Litoria dentata* is native to eastern Australia (coastal areas and adjacent uplands from southern New South Wales to southern Queensland, Fig 1), but was first recorded on LHI in 2002, by which time the population was “already well established” and by 2009, the species was found in all of the lowland areas (A. White, pers. comm.). *Litoria dentata* appears to be the only persistent amphibian species on LHI.

The Lord Howe Island Group (31°31’ S, 159°04’ E), is a small archipelago (~12 km x 2 km) of volcanic origin, that lies approximately 600 km east of the Australian mainland and 1400 km northwest of New Zealand. The LHI Group (incorporating LHI and associated islets) is a World Heritage Site and is considered a region of global ecological significance [21]. The LHI Group represents the remnants of an eroded shield volcano on the Lord Howe Rise in the South Pacific, that formed approximately 6.9 million years ago [22]. Most of the island’s steep sloped hills are covered in rainforest and the lowlands are generally forested [23]. It was one of the last places on Earth to be colonised, only being settled in 1833–1834 [24].

Habitat loss on LHI has been minimal, but many species have gone extinct or are currently threatened as a result of the arrival of invasive species [23,25,26]. Feral goats, pigs, cats, rats, mice and numerous species of plant have been introduced, either accidentally or deliberately, to LHI. These species have been implicated in the local extinction of some endemic species from the main island and the complete extinction of several other species from the entire LHI Group [25,27]. The impact of invasive species on LHI is significant and has been subjected to legislation to increase understanding of the non-native biota [21]. Knowledge of the basic biology and general ecology of an introduced species is necessary in order to determine the impact of an invasion. This information is also integral to making decisions about control and/or eradication of introduced species. The source and introduction history of the LHI population of *L*. *dentata* is unknown, but the frogs may have been introduced via either a sea (Yamba, Coffs Harbour) or air transport route (Sydney, Brisbane, Port Macquarie), or both, as observed for the invasive delicate skink, *Lampropholis delicata* [28].

Identifying the source of an invasive species provides vital information for biosecurity protocols and management and control of the introduced population. By determining the source region, the distinction of post-introduction evolution of the invasive population from plasticity within the native range can be assessed [29]. Additionally, differences between the two (or more) populations provide evolutionary models and the ability to detect founder effects, genetic bottlenecks and admixture [29,30]. Intentional species invasions are generally easier to investigate because the time, frequency and extent of the introduction have been documented. In contrast, identification of accidental invasion pathways is more complex, but essential to improving biosecurity measures [29]. Despite the known detrimental impact on novel ecosystems, many introductions of non-native organisms continue to take place, both accidentally and intentionally [31] and understanding introduction vectors and consequently altering biosecurity measures is a crucial step in preventing future introductions of amphibians to oceanic islands.

Here we use a mitochondrial phylogeographic approach, which has proved useful for offering reliable inference of source populations [18,32,33], to identify the source of the bleating tree frog, *Litoria dentata* on LHI. We then assess the biology and ecology of the introduced population, examining its distribution and habitat use across the island, as identified as a priority by the LHI Management Board [21]. Our study represents the first detailed study of *L*. *dentata* on LHI and outlines recommendations for biosecurity measures to prevent the accidental introduction of additional frog species to the island.

## Methods

### Identification of the source population(s) and introduction history

We collected tissue samples (toe clips—we did not remove whole animals as the frogs sampled were part of a broader mark-recapture study) from 36 *L*. *dentata* from across LHI during the 2010 and 2011 breeding seasons (S2 Table). Frogs were primarily caught from one of two breeding grounds (approximately 4 km apart), located at the northern (Old Settlement Beach) and central (Moseley Park) regions of LHI. In addition, we collected 22 tissue samples from mainland populations in eastern Australia (Ballina, Yamba, Grafton, Coffs Harbour), which were supplemented with 13 samples from the frozen tissue collection at the South Australian Museum (Australian Biological Tissue Collection; ABTC) collected throughout the native range of *L*. *dentata* (Fig 1). We included samples of the closely related species *Litoria rubella* and *Litoria electrica* as outgroups.

DNA was extracted from the tissue samples using the Qiagen DNeasy Blood and Tissue Extraction Kit (Qiagen, Hilden, Germany). To identify the source region(s), we amplified a portion of the ND4 mitochondrial gene for all LHI and native range samples (S2 Table) using the previously published primers ND4 and Limno2 [34]. This mitochondrial region has been used extensively in phylogenetic studies of *Litoria* species [35–39]. To further pinpoint the source locality we also sequenced the mitochondrial control region for a subset of LHI and native range samples (S2 Table), using the primers ControlJ2-L and ControlP-H [40]. PCR was conducted as outlined in Chapple et al. [41,42]. PCR products were purified using ExoSAP-IT (USB Corporation, Cleveland, OH, USA), and the purified product was sequenced by a commercial company (Macrogren) using a Big Dye Terminator v3.1 Cycle Sequencing Kit (Applied Biosystems, Carlsbad, CA, USA) and then analysed on an ABI 3730XL capillary sequencer. Sequence data were aligned and edited using the software Geneious R7 (created by Biomatters; available from http://www.geneious.com). For ND4, we translated all sequences in MEGA6 [43] to confirm that none contained premature stop codons. The haplotypes present in the ND4 and control region datasets were identified using DnaSP v5.10 [44] and were submitted to GenBank under accession numbers KM199696-KM199727 (S2 Table).

For the ND4 dataset, Maximum-Likelihood (ML) and Bayesian phylogenetic analyses were used to identify the native-range source region(s) for the introduction of *L*. *dentata* to LHI. We used jModeltest2 [45] to identify the most appropriate model of sequence evolution for our dataset. This model was used to generate a ML tree with 500 bootstraps using PhyML 3.0 [46,47]. The Bayesian analysis was completed using MrBayes 3.2.2 [48]. We ran the full analysis twice, using four Markov chains per run. We ran the chains for five million generations, to ensure sufficient sampling of tree space. The chain was sampled every 100 generations to obtain 50,000 sampled trees. The program Tracer v1.5 [49] was used to check for chain convergence. The first 25% of sampled trees were discarded as the burn-in phase and the last 37,500 trees were used to estimate the Bayesian posterior probabilities. Bootstrap values (500 ML bootstraps) and Bayesian posterior probabilities were used to assess branch support. For the control region dataset, we created a haplotype network in TCS v1.21 [50] to further pinpoint the native-range source region(s) for the introduction to LHI.

### Distribution, ecology and habitat use of *Litoria dentata* on Lord Howe Island

Initially, to determine the distribution of *L*. *dentata* across the island and identify habitat associations, time and space-constrained surveys were performed through stratified habitat (residential, native forest, pasture) along transects of 300 m in order to efficiently survey the island within the time available. However, very few frogs were encountered using this method and thus opportunistic searches were employed instead. Island-wide diurnal and nocturnal opportunistic searches were performed in July, September and December 2010 and December 2012. Natural cover objects were lifted and substrate and vegetation was searched with the aid of a head torch where necessary. There were no constraints on time or area searched. Frogs heard calling during searches were recorded, no other (inactive) frogs were encountered. All frogs included in this study were involved in breeding activity—calling, courting or mating.

For each frog captured, a tissue sample (toe clip) was taken and we recorded location, microhabitat, date, time and sex. Using digital callipers we measured (± 0.01 mm) snout-urostyle length, head width at widest point, head length, length of right femur, tibio-fibula, foot and radio-ulna. Frogs were weighed (± 0.01 g) using a pocket balance. We used independent sample, two-tailed t-tests to test for significance between sizes in males and females.

Calling surveys were carried out at 300 m intervals along roads and trails throughout the entire island. Observers stopped at each station in silence and without lights (if nocturnal survey) for two minutes before beginning the survey to reduce disturbance of frogs. Each survey lasted five minutes thereafter. The number of frogs calling at each station was recorded, as was their approximate location and habitat.

This research was conducted with the approval on the Monash University Animal Ethics Committee (Approval Number BSCI-2010-16) and in accordance with Australian (NSW scientific permits SL13154, SL100246; QLD Scientific permit WISP12166-12) research and collection permits.

## Results

### Identification of source population

**Fig 2:**
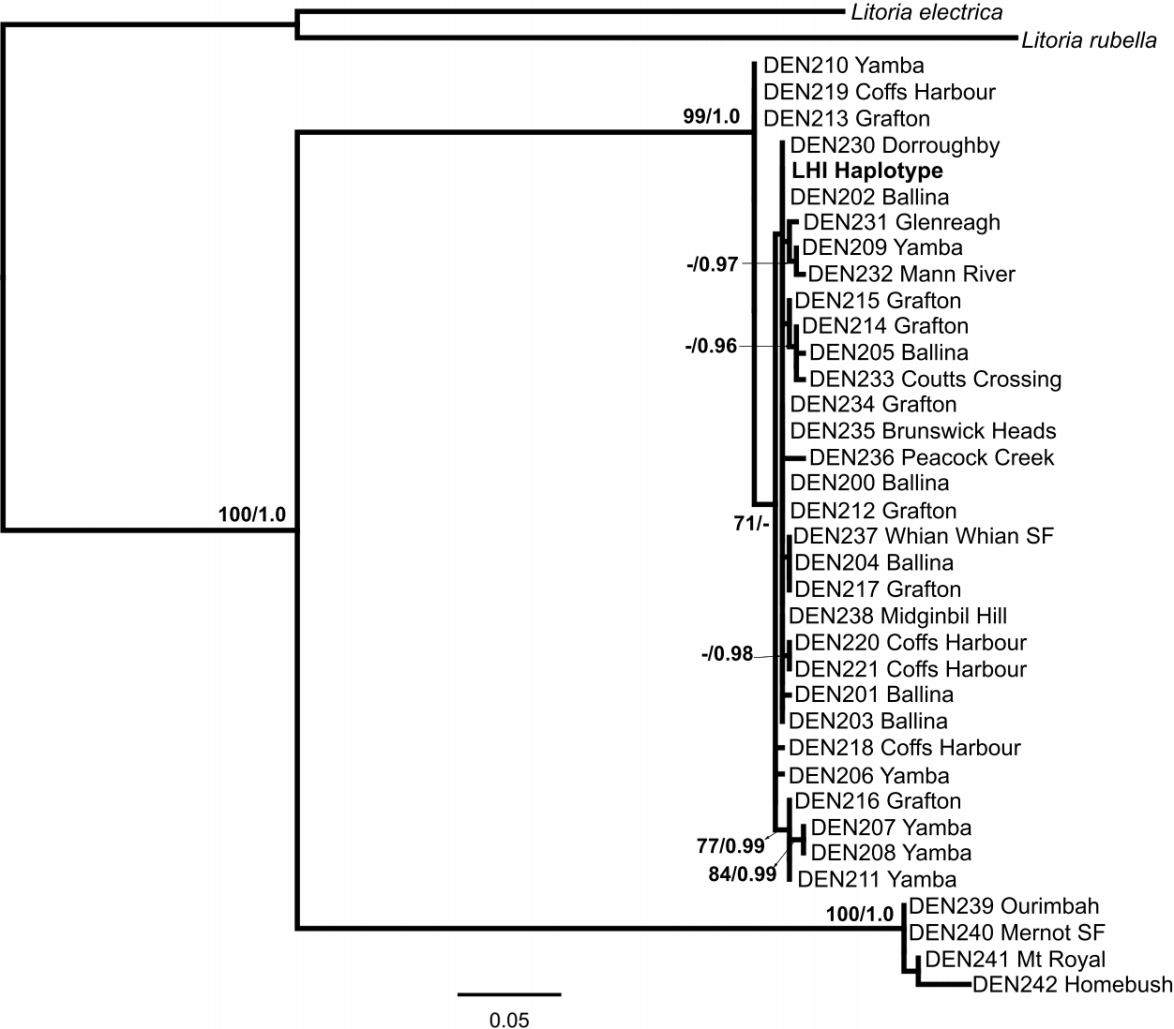
Maximum-Likelihood (ML) phylogram for *Litoria dentata* in Australia (native range and Lord Howe Island) based on ND4. Two major lineages are identified within *L*. *dentata*, roughly divided between the northern and southern portions of the native range. The Lord Howe Island population originates within the northern lineage. Two measures of branch support are indicated with ML bootstraps (500 replicates) on the left and Bayesian posterior probabilities on the right (only values over 50 and 0.7, respectively, are shown). The scale bar indicates branch length.

The ND4 alignment comprised 655 bp, with 223 (34.0%) variable and 148 (22.6%) parsimony-informative sites. For the ingroup, there were 122 (18.6%) variable characters, of which 111 (16.9%) were parsimony-informative. The base frequencies were A = 0.272, T = 0.344, C = 0.247, G = 0.138). The Bayesian Information Criteria (BIC) from jModeltest2 supported the HKY + I substitution model for the ND4 dataset. Parameters estimated under this model were: relative substitution rates (A↔C = 1.4071, A↔G = 7.8932, A↔T = 1.8624, C↔G = 0.5571, C↔T = 7.6022, relative to G↔T = 1.0000), and proportion of invariable sites (0.544). The topology of the ML and Bayesian trees were almost identical, and thus, we present the optimal ML tree (-ln *L* = 2191.419) with ML bootstrap (BS) values and Bayesian posterior probabilities (PP) indicating branch support (Fig 2).

While *L*. *dentata* represents a monophyletic lineage (100 BS, 1.0 PP), two divergent genetic subclades were evident: a southern clade (100 BS, 1.0 PP) and a northern clade (99 BS, 1.0 PP) (Fig 2). All LHI individuals shared the same ND4 haplotype, which fell into the northern clade (Fig 2). The LHI ND4 haplotype was found also in Ballina (DEN200, DEN202, DEN203), Grafton (DEN212, DEN234), Dorroughby (DEN230), Midginbil (DEN238), and Brunswick Heads (DEN235) (Fig 2, S2 Table).

**Fig 3:**
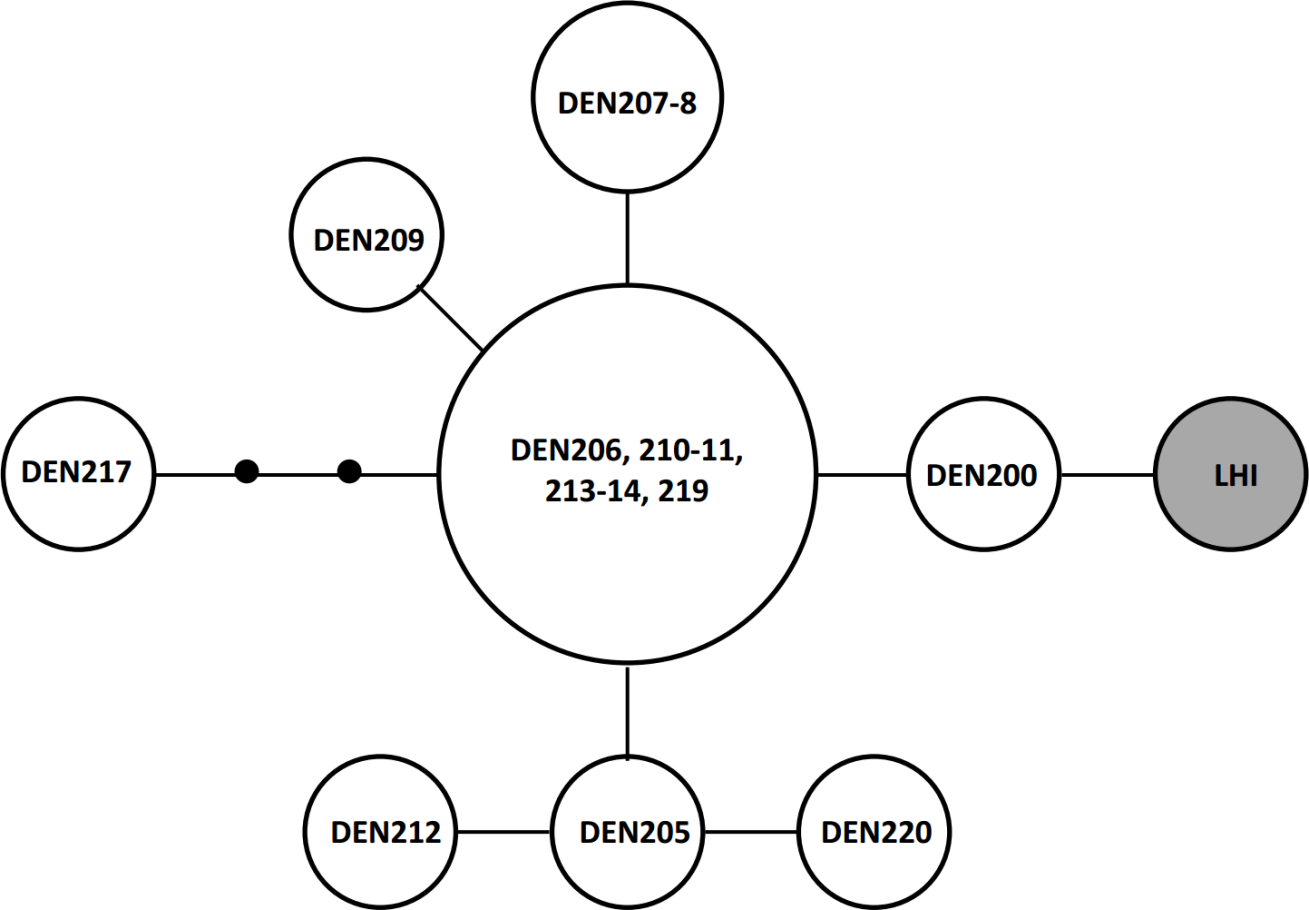
Control region haplotype network for *Litoria dentata*. Each circle represents one haplotype and the size indicates the number of individuals with each haplotype. The lines indicate single mutations between haplotypes, and the dots indicate potential haplotypes that were not observed in the study. Letters and numbers refer to the sample identification code.

The control region dataset (396 bp, 10 variable sites [2.5%], 3 parsimony-informative sites [0.8%]; base frequencies A = 0.330, T = 0.413, C = 0.100, G = 0.157) indicated that all LHI samples had the same haplotype (Fig 3). There was no exact native range match for this haplotype, but it was only 1 mutation away from a sample from Ballina (DEN200).

### Biology of *Litoria dentata* on Lord Howe Island

**Fig 4:**
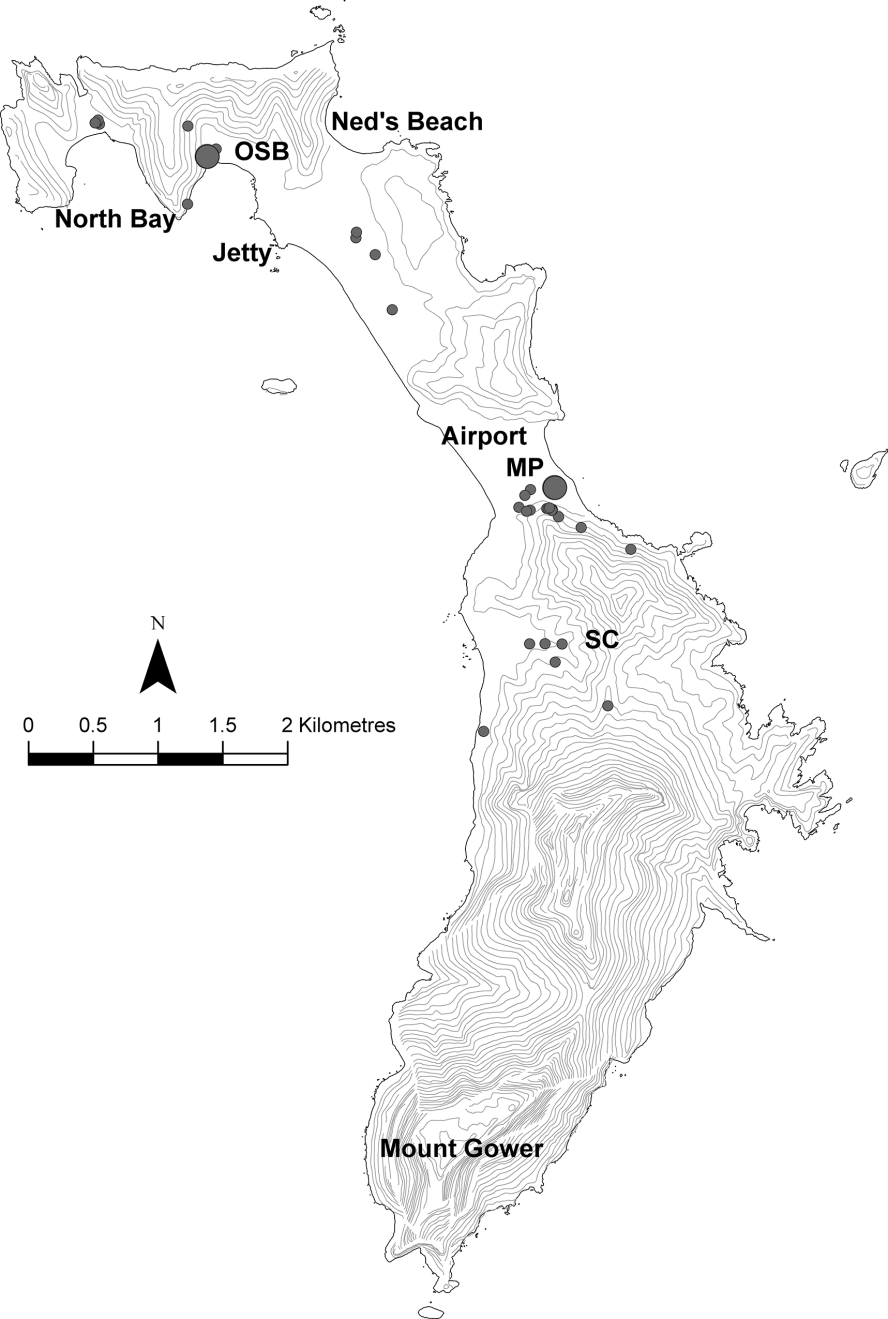
Map showing locations on Lord Howe Island from where *Litoria dentata* were found. Small circles represent <5 individuals, large circles represent >17 individuals. OSB = Old Settlement Beach, MP = Moseley Park, SC = Soldier’s Creek area.

*Litoria dentata* is widely distributed across much of LHI (Fig 4). It occurs in high densities in the central and northern regions but is concentrated around breeding areas (i.e., flooded pastures close to the settlement). Of the 178 frogs recorded on LHI by visual or auditory observations, 165 were found within or in close proximity to the settlement area, although this is likely an artefact of sampling regime. The majority of frogs (116) found were observed during opportunistic searches and were located in or immediately surrounding the water at breeding grounds. These were all detected outside of the time and space-constrained sampling regime. One frog was found within human habitation, two were collected from palm fronds in a forested area and 56 were recorded calling in or on trees. All but one frog heard during call surveys could not be physically located and were determined to be within trees. One frog was collected dead, discovered within rolls of timber recently delivered to the island and two frogs were collected recorded without microhabitat data. Island residents have reported the frogs in human dwellings and water vessels. Most, if not all, terrestrial habitats on LHI are both suitable and being utilised by *L*. *dentata*. We did not record frogs in the very southernmost portion of LHI (Mount Gower).

A total of six individuals were observed during the calling surveys, the other 50 auditory observations were recorded during the opportunistic searches. *Litoria dentata* frequently calls from trees within a few hundred metres of known breeding grounds on LHI. Most frogs were encountered during periods of heavy rainfall. Calling activity appeared to increase in periods of rising humidity levels and as such, occurred at any time during the day or night.

A total of 87 males and 35 females were captured on LHI, mostly during breeding events. Only mature animals were measured live (66 males and 26 females are included in the analysis) to prevent confounding effects of shrinking in preservative. Often it was not possible to detect the reproductive status of females, but most were presumed to be gravid as activity seems largely to be restricted to reproductive periods. Males were significantly smaller than females, but only in SUL, tibio-fibula length and foot length (Table 1). There was no significant difference in head width, head length, femur length, radio-ulna length and weight. Two amplecting pairs were found and in both, amplexus was axillary.

**Table 1:** Mean morphology of Lord Howe Island *Litoria dentata*.

	SUL	Head width	Head length	Femur length	Tibio-fibula length	Foot length	Radio-ulna length	Weight
Males (n = 66)	36.32 (± 1.59) ***	10.68 (± 0.58)	10.75 (± 1.02)	14.70 (± 1.16)	15.53 (± 0.72) *	9.30 (± 0.59)	6.68 (±0.74)	2.88 (± 0.47)
Females (n = 26)	38.48 (± 2.30) ***	10.89 (± 0.61)	11.11 (± 1.26)	15.18 (± 1.46)	16.16 (± 1.19)*	9.66 (± 0.79)	6.89 (± 0.56)	3.16 (± 0.76)

Measurements are in mm for lengths and g for weight (± SD). *Alpha* levels 0.05 and 0.001 are indicated by * and *** respectively for differences between males and females.

## Discussion

### *Litoria dentata* was introduced to Lord Howe Island from Northern New South Wales

The source region of the LHI population of *L*. *dentata* was identified as northern New South Wales, in the Ballina/Yamba area. The presence of only a single haplotype on LHI is consistent with either a single introduction event, or from multiple introduction events from the same source population. Thus, the species was most likely introduced to the island on the supply ship that serviced the island from the Yamba region during the 1980s and 1990s, when the frog was first noted by residents on LHI. This also corresponds with reports from LHI residents that frogs arrived with shipments of vegetables. The delicate skink (*Lampropholis delicata*) has also become invasive on LHI after multiple successful (accidental) introductions, including shipment through Yamba from the northern NSW region [28]. The paucity of biosecurity precautions on this trade route is clearly a risk factor in unintentional transport of non-native species to LHI.

Three lineages were identified in the native range, of which only one was found to be on LHI. The presence of only one haplotype in the LHI population of *L*. *dentata* suggests that it may have undergone a substantial founder effect. The lack of genetic diversity is likely to negatively affect the LHI population by reducing potential for adaptation and limiting population growth [51]. However, genetic architecture may be more important than a large gene pool [52] and persistence of the species on LHI is dependent on sufficient diversity in the island population. Other invasive frogs have been known to have successfully invaded similar environments, despite genetic bottlenecks [53], some with tiny founder populations [54]. A species of European newt has recently been documented to be persisting and spreading in its introduced range in Australia, despite also only being represented by a single haplotype [55]. In addition, the importance of the presence of multiple lineages has often been overemphasised in the past (reviewed in Chapple et al. 2013 [28]). The successful reproduction and spread of *L*. *dentata* on LHI over at least 25 years indicates a high chance of persistence into the future.

### Distribution, habitat use and morphology of *Litoria dentata* on Lord Howe Island

*Litoria dentata* is distributed across most of LHI. This broad range throughout many of the island’s wetlands has wide-reaching implications if the frogs are detrimental to the island’s ecosystems. The greatest numbers of *L*. *dentata* were detected in temporary wetlands at Old Settlement Beach and Moseley Park and frog presence and activity and hydrologic regimes in other areas suggest that it is likely the frogs are breeding in low lying regions elsewhere. Previous observations (A. White, pers. comms.) also include a report of *L*. *dentata* at Ned’s Beach, additional to our records. We did not record frogs on Mount Gower; we did not survey this area as extensively as the other parts of the island due to accessibility and logistical issues. However, island residents report hearing them and it is likely that they will soon infiltrate the forests of the southernmost slopes of LHI, if they have not already. Incidental encounters proved the most useful method of distribution and habitat data collection. Stratified surveys were of limited use due to the specific and narrow requirements for activity of *L*. *dentata* (our unpublished data). Calling surveys probably are not reliable indicators of frog distribution due to low detectability in most conditions, but the size and terrain of the island and the large distribution of samples means we are confident that *L*. *dentata* is widespread and abundant across LHI.

Almost all the individuals encountered on LHI were mature adults. This is likely due to the increase in activity of breeding adults and a short developmental period, lowering detectability of smaller size classes. The explosive breeding (short–term, synchronised aggregated breeding) trait and lekking produced an uneven sex ratio as an artefact of sampling bias for calling males which are likely to arrive at the pond earlier, spend more time in exposed positions and attract attention with their noise [56]. *Litoria dentata* are documented to breed in ephemeral wetlands in disturbed, sub-optimal habitat [57]. This is consistent with the other species of invasive *Litoria* which reproduce in flooded areas with high levels of anthropogenic disturbance in their native and introduced range.

The individuals captured and measured on LHI were smaller than other populations of *L*. *dentata*, which are largely cited as approximately 45 mm (e.g., [58]). A population measured in Sydney, as with the LHI frogs, also presented sexual size dimorphism, but consisted of larger animals (male mean snout-vent length 39.6mm, female mean snout-vent length 42.1 mm [57]).

### Invasion success in *Litoria*

*Litoria* frogs have been introduced through much of the South Pacific. The species that have become invasive are often habitat generalists that breed in temporary open waters, increasing their ability to adapt to novel environments ([13], S1 Table). The explosive breeding system common to these species is characterised by high densities of individuals during reproductive events, which would likely increase the chances of large numbers being transported by anthropogenic routes to a new locality in any single event. Breeding aggregations also decrease the reduction of fitness caused by a small population size (i.e., Allee effect) by increasing the chances of an individual finding a mate and improving invasion success [59]. It is likely that the introduction of *L*. *dentata* to LHI includes a one-off movement when animals became active in the vicinity of the goods and breeding adults, egg masses or tadpole clutches were transported to the island. The introduction must have consisted of sufficient numbers to ensure reproductive success once on the island. Spread of the LHI population was probably assisted by transport of goods around the island.

### Biosecurity on Oceanic Islands: Lord Howe Island as a case study

Recognising the common traits of the most frequent animal invaders can help direct interception of invading species. The frog family Hylidae represents a disproportionate number of successful amphibian invasions [60]. This taxonomic bias presents the opportunity to target potential invaders during the early stages of invasion, transport and introduction. The ability to predict potential invasive species and transportation pathways could be used to further assist the prevention of introductions. The delivery of goods to LHI represents many risk factors that increase the chance of uptake and transport as the cargo is shipped from temperate, low elevation areas with dense human populations and habitat similar to LHI which significantly increases the chances of bringing non-native species with it [60].

Before the settlement of LHI, many endemic invertebrates inhabited the island and the high abundance of an insectivorous frog will likely impact the invertebrate community, as has been observed with other invasive anurans [61–63]. *Litoria dentata* also likely acts as a food source for the birds that frequent the ponds in which the frog breeds on LHI, such as white-faced herons (*Egretta novaehollandiae*) and currawongs (*Strepera graculina*). Furthermore, the species is known for its loud call and has the potential to make a social impact [64] as it is found in and around residences and the explosive breeding trait is associated with large numbers of calling males. Invasive frogs in Hawai’i have been implicated in the reduction of property value and economic effects due to their undesirable levels of noise [65]. As is also seen with the coqui frog, *Eleutherodactylus coqui*, on Hawaiian islands, *L*. *dentata* may also impact the palm nursery industry if people fear moving the frogs via the plants [66]. Given the abundance and distribution of *L*. *dentata* on LHI, it is feasible that the species is responsible for some impact on the island ecosystem, although the quantification of such an impact is beyond the scope of this paper. Regardless of quantifiable impact of *L*. *dentata* specifically, our study suggests that stronger biosecurity procedures may be necessary to help protect LHI from future invasions by other species.

The introduction of *L*. *dentata* to the island highlights potential problems with biosecurity measures on trade routes between mainland Australia and LHI and has serious implications for the potential for introduction of other species, such as the cane toad (*Rhinella marina*) or Asian house gecko (*Hemidactylus frenatus*). The invasion of *L*. *dentata* to LHI was unintentional and similar to that of *L*. *delicata*, introduced to LHI in a similar time period. The skink was introduced on multiple occasions [28] and likely with cargo shipped from mainland Australia. This demonstrates serious flaws in biosecurity measures on LHI. One introduced haplotype of the LHI population of *L*. *delicata* originated in northern New South Wales within close proximity to the source population of *L*. *dentata*, indicating an increased chance of further invasions via the same pathway. We recommend minimising opportunities for transport by improving housing and restrictions for cargo at the port before it leaves the mainland and recognising sources of introductions and high risk cargo (e.g., fresh produce) and demonstrating greater vigilance with such material. Suppliers should be selected according to their capacity for invasive species risk assessment and quarantine procedures. Once on LHI, comprehensive screening and stringent removal of stowaway fauna in cargo and produce before it is unloaded and dispersed over the island would reduce incidence of introductions.

## Supporting Information

S1 Table Ecological features of invasive *Litoria* species in the Pacific region. (DOC)

S2 Table Collection localities on Lord Howe Island and in Eastern Australia of *Litoria dentata* samples. (DOC)

## References

[1] Macarthur RH, Wilson EO. The Theory of Island Biogeography. Princeton, New Jersey: Princeton University Press; 1967.

[2] Whittaker RJ, Fernandez-Palacios JM. Island Biogeography: Ecology, Evolution, and Conservation. Oxford: Oxford University Press; 2007.

[3] Vitousek PM. Diversity and biological invasions of oceanic islands In: Wilson EO, editor. Biodiversity. Washington, D.C.: National Academy Press; 1988 p. 181–9.

[4] Measey GJ, Vences M, Drewes RC, Chiari Y, Melo M, Bourles B. Freshwater paths across the ocean: molecular phylogeny of the frog Ptychadena newtoni gives insights into amphibian colonization of oceanic islands. Journal of Biogeography. 2007;34(1):7–20.

[5] Gillespie GR, Roderick GK. Arthropods on islands: Colonization, speciation, and conservation. Annual Review of Entomology 2002;47:595–632. 11729086 10.1146/annurev.ento.47.091201.145244

[6] Vidal N, Azvolinsky A, Cruaud C, Hedges SB. Origin of tropical American burrowing reptiles by transatlantic rafting. Biology Letters. 2008 2;4(1):115–8. PubMed . English. 18077239 10.1098/rsbl.2007.0531 PMC2412945

[7] Cowie RH, Holland BS. Molecular biogeography and diversification of the endemic terrestrial fauna of the Hawaiian Islands. Philosophical Transactions of the Royal Society B-Biological Sciences. 2008 10 27;363(1508):3363–76. PubMed 10.1098/rstb.2008.0061 PMC2607369 18765363

[8] Paulay G. Biodiversity on oceanic islands—its origin and extinction. American Zoologist. 1994 1994;34(1):134–44. PubMed .

[9] Reaser JK, Meyerson LA, Cronk Q, De Poorter M, Eldrege LG, Green E, et al Ecological and socioeconomic impacts of invasive alien species in island ecosystems. Environmental Conservation. 2007 6;34(2):98–111. PubMed .

[10] Sugiura S. Species interactions-area relationships: biological invasions and network structure in relation to island area. Proceedings of the Royal Society B-Biological Sciences. 2010 6 22;277(1689):1807–15. PubMed 10.1098/rspb.2009.2086 PMC2871870 20147330

[11] Blackburn TM, Cassey P, Duncan RP, Evans KL, Gaston KJ. Avian extinction and mammalian introductions on oceanic islands. Science. 2004 9 24;305(5692):1955–8. PubMed . 15448269 10.1126/science.1101617

[12] Lever C. Naturalized Reptiles and Amphibians of the World. New York: Oxford University Press; 2003.

[13] Kraus F. Alien Reptiles and Amphibians: A Scientific Compendium and Analysis. Drake JA, editor: Springer; 2009.

[14] McKeown S. Notes on a newly established frog, *Eleutherodactylus coqui*, in the Haiwaiian Islands. Bulletin of the Chicago Herpetological Society. 1998;33(30–31).

[15] Kraus F, Campbell EW, Allison A, Pratt T. *Eleutherodactylus* frog introductions to Hawaii. Herpetological Review. 1999;30(1):21–5.

[16] Kraus F, Campbell EW. Human-mediated escalation of a formerly eradicable problem: the invasion of Caribbean frogs in the Hawaiian Islands. Biological Invasions. 2002;4:327–32.

[17] Tyler MJ. Introduction and current distribution in the New-Hebrides of the Australian Hylid frog *Litoria aurea* . Copeia. 1979 (2):355–6. PubMed .

[18] Vörös J, Mitchell A, Waldman B, Goldstein S, Gemmell NJ. Crossing the Tasman Sea: Inferring the introduction history of *Litoria aurea* and *Litoria raniformis* (Anura: Hylidae) from Australia into New Zealand. Austral Ecology. 2008;33:623–9.

[19] Bell BD, Carver S, Mitchell NJ, Pledger S. The recent decline of a New Zealand endemic: how and why did populations of Archey's frog Leiopelma archeyi crash over 1996–2001? Biological Conservation. 2004;120(2):189–99.

[20] Anon. Lord Howe Island Permanent Park Preserve Plan of Management. In: Department of the Environment CCaWN, editor.: Lord Howe Island Board; 2010.

[21] Lord Howe Island Biodiversity Management Plan. In: (NSW) DoEaCC, editor. Sydney: Department of Environment and Climate Change (NSW); 2007.

[22] McDougall I, Embleton BJJ, Stone DB. Origin and evolution of Lord Howe Island, Southwest Pacific Ocean. Australian Journal of Earth Sciences. 1981;28(1):155–76.

[23] Recher HF, Clark SS. A biological survey of Lord Howe Island with recommendations for the conservation of the island's wildlife. Biological Conservation. 1974;6(4):263–73.

[24] Nicholls M. A history of Lord Howe Island: Printed by Mercury Press; 1952.

[25] Hutton I, Parkes JP, Sinclair ARE. Reassembling island ecosystems: the case of Lord Howe Island. Animal Conservation. 2007 2;10(1):22–9. PubMed .

[26] Auld TD, Hutton I, Ooi MKJ, Denham AJ. Disruption of recruitment in two endemic palms on Lord Howe Island by invasive rats. Biological Invasions. 2010 9;12(9):3351–61. PubMed .

[27] Hindwood KA. The birds of Lord Howe Island. Emu. 1940;40(1):1–86.

[28] Chapple DG, Miller KA, Kraus F, Thompson MB. Divergent introduction histories among invasive populations of the delicate skink (Lampropholis delicata): has the importance of genetic admixture in the success of biological invasions been overemphasized? Diversity and Distributions. 2013 2;19(2):134–46. PubMed .

[29] Estoup A, Guillemaud T. Reconstructing routes of invasion using genetic data: why, how and so what? Molecular Ecology. 2010 10;19(19):4113–30. PubMed 10.1111/j.1365-294X.2010.04773.x 20723048

[30] Dlugosch KM, Parker M. Founding events in species invasions: genetic variation, adaptive evolution, and the role of multiple introductions. Molecular Ecology. 2008;17:431–49. 17908213 10.1111/j.1365-294X.2007.03538.x

[31] Lockwood JL, Hoopes MF, Marchetti MP. Invasion Ecology. Second ed. Malden, Massachusetts, USA: Wiley-Blackwell; 2013.

[32] Corin SE, Lester PJ, Abbott KL, Ritchie PA. Inferring historical introduction pathways with mitochondrial DNA: the case of introduced Argentine ants (*Linepithema humile*) into New Zealand. Diversity and Distributions. 2007;13:510–8.

[33] Muirhead JR, Gray DK, Kelly DW, Ellis SM, Heath DD, MacIsaac HJ. Identifying the source of species invasions: sampling intensity vs. genetic diversity. Molecular Ecology. 2008;17:1020–35. 10.1111/j.1365-294X.2008.03669.x 18261046

[34] Arevalo E, Davis SK, Sites JW. Mitochondrial DNA sequence divergence and phylogenetic relationships among eight chromosome races of the Sceloporus grammicus complex (Phrynosomatidae) in Central Mexico. Systematic Biology. 1994 9;43(3):387–418. PubMed .

[35] Schäuble CS, Moritz C. Comparative phylogeography of two open forest frogs from eastern Australia. Biol J Linnean Soc. 2001;74(2):157–70.

[36] Mahony M, Knowles R, Foster R, Donnellan SC. Systematics of the Litoria citropa (Anura: Hylidae) complex in northern New South Wales and southern Queensland, Australia, with the description of a new species. Records-Australian Museum. 2001;53(1):37–48.

[37] Burns EL, Crayn DM. Phylogenetics and evolution of bell frogs (< i> Litoria aurea species-group, Anura: Hylidae) based on mitochondrial< i> ND4 sequences. Molecular Phylogenetics and Evolution. 2006;39(2):573–9. 16384717 10.1016/j.ympev.2005.11.017

[38] Burns EL, Eldridge MD, Crayn DM, Houlden BA. Low phylogeographic structure in a wide spread endangered Australian frog *Litoria aurea* (Anura: Hylidae). Conservation Genetics. 2007;8(1):17–32.

[39] Smith KL, HJ M., Kearney MR, Austin JJ, Melville J. Molecular patterns of introgression in a classic hybrid zone between the Australian tree frogs, *Litoria ewingii* and *L*. *paraewingi*: evidence of a tension zone. Molecular Ecology. 2013;22(7):1869–83. 10.1111/mec.12176 23294099

[40] Goebel AM, Donnelly JM, Atz ME. PCR primers and amplification methods for 12S ribosomal DNA, the control region, cytochrome oxidase I, and cytochrome b in bufonids and other frogs, and an overview of PCR primers which have amplified DNA in amphibians successfully. Molecular Phylogenetics and Evolution. 1999;11(1):163–99. 10082619 10.1006/mpev.1998.0538

[41] Chapple DG, Hoskin CJ, Chapple SNJ, Thompson MB. Phylogeographic divergence in the widespread delicate skink (Lampropholis delicata) corresponds to dry habitat barriers in eastern Australia. Bmc Evolutionary Biology. 2011 7 4;11 PubMed . 10.1186/1471-2148-11-191 PMC3141439 21726459

[42] Chapple DG, Bell TP, Chapple SNJ, Miller KA, Daugherty CH, Patterson GB. Phylogeography and taxonomic revision of the New Zealand cryptic skink (Oligosoma inconspicuum; Reptilia: Scincidae) species complex. Zootaxa. 2011 3 3(2782):1–33. PubMed .

[43] Tamura K, Stecher G, Peterson D, Filipski A, Kumar S. MEGA6: Molecular Evolutionary Genetics Analysis version 6.0. Molecular Biology and Evolution. 2013;30:2725–9. 10.1093/molbev/mst197 24132122 PMC3840312

[44] Librado P, Rozas J. DnaSP v5: A software for comprehensive analysis of DNA polymorphism data. Bioinformatics. 2009;25:1451–2. 10.1093/bioinformatics/btp187 19346325

[45] Darriba D, Taboada GL, Doallo R, Posada D. jModelTest 2: more models, new heuristics and parallel computing. Nature Methods. 2012;9(8):772 10.1038/nmeth.2109 22847109 PMC4594756

[46] Guindon S, Gascuel O. A simple, fast and accurate method to estimate large phylogenies by maximum-likelihood. Systematic Biology. 2003;52:696–704. 14530136 10.1080/10635150390235520

[47] Guindon S, Dufayard JF, Lefort V, Anisimova M, Hordijk W, Gascuel O. New algorithms and methods to estimate maximum-likelihood phylogenies: assessing the performance of PhyML 3.0. Systematic Biology. 2010;59:307–21. 10.1093/sysbio/syq010 20525638

[48] Ronquist F, Huelsenbeck J, Teslenko M. Draft MrBayes version 3.2 manual: tutorial and model summaries. 2011. Available: http://mrbayes.sourceforge.net/mb3.2_manual.pdf. Accessed 17 July 2014.

[49] Rambaut A, Drummond A. Tracer v1.5 http://tree.bio.ed.ac.uk2007 [17th July 2014].

[50] Clement M, Posada D, Crandall KA. TCS: a computer program to estimate gene genealogies. Molecular Ecology. 2000;9:1657–9. 11050560 10.1046/j.1365-294x.2000.01020.x

[51] Sakai AK, Allendorf FW, Holt JS, Lodge DM, Molofsky J, With KA, et al The population biology of invasive species. Annual Review of Ecology and Systematics. 2001;32:305–32. PubMed .

[52] Lee CE. Evolutionary genetics of invasive species. Trends in Ecology & Evolution. 2002 8;17(8):386–91. PubMed .

[53] Peacock MM, Beard KH, O'Neill EM, Kirchoff VS, Peters MB. Strong founder effects and low genetic diversity in introduced populations of Coqui frogs. Molecular Ecology. 2009 9;18(17):3603–15. PubMed 10.1111/j.1365-294X.2009.04308.x 19674300

[54] Ficetola GF, Bonin A, Miaud C. Population genetics reveals origin and number of founders in a biological invasion. Molecular Ecology. 2008;17(3):773–82. 10.1111/j.1365-294X.2007.03622.x 18194168

[55] Tingley R, Weeks AR, Smart AS, van Rooyen AR, Woolnough AP, McCarthy MA. European newts establish in Australia, marking the arrival of a new amphibian order. Biological Invasions. 2015;17(1):31–7.

[56] Halliday T. Ecological Census Techniques: A Handbook, 2nd Edition Cambridge: Cambridge University Press; 2006. 1–432 p.

[57] Greer AE, Mills A. Observations on the biology of the Bleating Tree Frog *Litoria dentata* (Anura: Hylidae), made on a single population in Sydney, New South Wales. Australian Zoologist. 1998;30(4):383–6.

[58] Cogger HG. Reptiles and amphibians of Australia 7 ed. Melbourne: CSIRO Publishing; 2014.

[59] Salinas FV. Breeding behavior and colonization success of the Cuban treefrog Osteopilus septentrionalis. 2009.

[60] Tingley R, Romagosa CM, Kraus F, Bickford D, Phillips BL, Shine R. The frog filter: amphibian introduction bias driven by taxonomy, body size and biogeography. Global Ecology and Biogeography. 2010 7;19(4):496–503. PubMed .

[61] Beard KH, Eschtruth AK, Vogt KA, Vogt DJ, Scatena FN. The effects of the frog Eleutherodactylus coqui on invertebrates and ecosystem processes at two scales in the Luquillo Experimental Forest, Puerto Rico. Journal of Tropical Ecology. 2003 11;19:607–17. PubMed . English.

[62] Shine R. The ecological impact of invasive cane toads (*Bufo marinus*) in Australia. Quarterly Review of Biology. 2010 9;85(3):253–91. PubMed . 20919631 10.1086/655116

[63] Choi RT, Beard KH. Coqui frog invasions change invertebrate communities in Hawaii. Biological Invasions. 2012 5;14(5):939–48. PubMed .

[64] Kaiser BA, Burnett K. Economic impacts of *E*. *coqui* frogs in Hawaii. Interdisciplinary Environmental Review. 2006;8(2):1–11.

[65] Beard KH, Pitt WC. Potential consequences of the coqui frog invasion in Hawaii. Diversity and Distributions. 2005 9;11(5):427–33. PubMed .

[66] Beard KH, Price EA, Pitt WC. Biology and Impacts of Pacific Island Invasive Species. 5. Eleutherodactylus coqui, the Coqui Frog (Anura: Leptodactylidae). Pacific Science. 2009 7;63(3):297–316. PubMed 10.1007/s11418-009-0335-7

