# Supplementary material for: Human-Assisted Invasions of Pacific Islands by Litoria Frogs: A Case Study of the Bleating Tree Frog on Lord Howe Island
Source: PLoS One. 2015 May 11;10(5):e0126287. doi: 10.1371/journal.pone.0126287 (PMC4427294; doi:10.1371/journal.pone.0126287)
Supplement: S1 Table — (DOC) [file pone.0126287.s001.doc]

**Supporting Information Table S1: Ecological features of invasive *Litoria* species in the Pacific region.**

| Species | Native area | Invasive locality | Introduction | Reproductive ecology | Habitat use | References |
| --- | --- | --- | --- | --- | --- | --- |
| *aurea* | Eastern Australia  Vulnerable in its native range [1] | New Zealand,  Loyalty Islands,  New Caledonia,  Vanuatu,  Wallis Island | Deliberate*  Introduced to New Caledonia prior to 1890, presumably from Australia, but may have been North Island New Zealand. | Breeds in open wetlands (ponds, lakes, dams etc.).  Loud choruses September-February from middle of pond in New Zealand and from surrounding vegetation on other islands.  Pectoral amplexus.  Free-feeding, active tadpoles. | Shelters under natural cover objects and in vegetation.  Found in most habitats on islands. Common in disturbed lowland sites such as gardens. Individual males call from ground cover or low branches in native forests. | [2-32] |
| *dentata* | Eastern Australia | Lord Howe Island | Unintentional | Breeds in open, mostly temporary wetlands during and after very heavy rain.  May call from high in trees during periods of high humidity. | Shelters in tree hollows away from breeding grounds. Often found in human habitation and infrastructure such as water tanks. | This paper |
| *ewingi* | Tasmania | New Zealand | Introduced to Greymouth by W. Perkins 1875 | Breeds in open wetlands with loud choruses, mostly at night and year-round.  Calls from vegetation at and away from pond’s edge, summer and winter, most often at night close to shore, but even in the daytime from dry scrub.  Breeds in more shallow and temporary pools and puddles.  Amplexus pectoral.  Active, free-feeding tadpoles. | Hides or basks in herbaceous vegetation such as grasses, sedges, reeds and flax, low scrub, under stones or logs or several metres off the ground in trees.  Independent of water when not breeding.  Coastal and lowland and above the tree line in some areas. | [2,3,5,9,10,19,21,23,28,29,32-38] |
| *fallax (glauerti)* | Australia | Guam | Stowaway prior to 1968 | Breeds in open wetlands with submerged vegetation, onto which eggs are attached.  Calls from vegetation around ponds in Spring and Summer. | Gardens, clear farmland and open forests. | [2,12,15,16,32,39,40] |
| *raniformis* | Tasmania  Endangered in its native range [41] | New Zealand | Deliberate† | Breeds spring and early summer in open wetlands (ponds, lakes, dams etc.).  Loud choruses, August to February. Calls from surface of middle of pond.  Amplexus pectoral.  Active, free-feeding tadpoles  Completes metamorphosis in shallow, probably temporary pools. | Shelters under stones, logs, clumps of vegetation or similar.  Found under driftwood on the upper shore at edge of brackish pools and under cover on forest floor at higher altitudes. | [2,9,10,14,20,22,28-30,33,37,38,42,43] |

*Introduced to New Zealand by Auckland Acclimatisation Society in 1867/1868 † Introduced to New Zealand by Canterbury Acclimatisation Society in 1867

1. Hero JM, Gillespie GR, Cogger HG, Lemckert F, Robertson P (2004) *Litoria aurea* In: IUCN 2014. IUCN Red List of Threatened Species Version 3.1. http://www.iucnredlist.org. Accessed 18 March 2015.

2. Lever C (2003) Naturalized Reptiles and Amphibians of the World. New York: Oxford University Press.

3. Archey G (1935) Frogs in New Zealand. Bulletin of the Auckland Zoological Society 2: 5-8.

4. Ash LR (1968) The occurence of *Angiostrongylus cantonensis* in frogs of New Caledonia with observations on paratenic hosts of metastrongyles. Journal of Parasitology 54: 432-436.

5. Barwick RE (1961) Illustrations of the New Zealand frog fauna. Tuatara 8: 95-98.

6. Bauer AM, Sadlier RA, Chazeau J (1997) Geographic distribution: *Litoria aurea*. Herpetological Review 28: 156.

7. Bauer AM, Sadlier RA (2000) The herpetofauna of New Caledonia. Ithaca, NY: Society for the Study of Amphibians and Reptiles.

8. Bauer AM, Vindum JV (1990) A checklist and key to the herpetofauna of New Caledonia, with remarks on biogeography. Proceedings of the California Academy of Sciences 47: 17-45.

9. Bell BD (1982) The amphibian fauna of New Zealand.

10. Bell BD (1982) New Zealand Frogs. Herpetofauna 14: 1-21.

11. Bishop PJ (1999) Declining frog populations in New Zealand - the New Zealand Frog Survey and possible future directions. New Zealand Journal of Zoology 26: 255-256.

12. Christy MT, Clark CS, Gee DE, II, Vice D, Vice DS, et al. (2007) Recent records of alien anurans on the Pacific Island of Guam. Pacific Science 61: 469-483.

13. Colgan D (1996) Electrophoretic variation in the green and golden bell frog *Litoria aurea*. Australian Zoologist 30: 170-176.

14. Copland SJ (1957) Presidential Address. Australian tree frogs of the genus *Hyla*. Proceedings of the Linnean Society of New South Wales 82: 9-108.

15. Eldredge LG (1988) Case studies of the impacts of introduced animal species on renewable resources in the U.S.-affiliated Pacific islands. University of Guam Marine Laboratory. 26-46 p.

16. Eldredge LG (2000) Non-indigenous freshwater fishes, amphibians, and crustaceans of the Pacific and Hawaiian islands; Sherley G, editor. Apia, Samoa: South Pacific Regional Environment Programme.

17. Ford MA (1989) Vocalisations of the green frogs *Litoria aurea* and *Litoria raniformis*, including species-specific male calls. New Zealand Journal of Zoology 16: 17-23.

18. Gargominy O, Bouchet P, Pascal M, Jaffré T, Tourneur J-C (1996) Conséquences des introctions d'espéces animales et végétales sur la biodiversité en Nouvelle-Calédonie. Revue d'Ecologie la Terre et al Vie 51: 375-402.

19. Gill B (1986) Collins handguide to the frogs and reptiles of New Zealand. Auckland: Collins.

20. Gill BJ, Bejakovich D, Whitaker AH (2001) Records of foreign reptiles and amphibians accidentally imported to New Zealand. New Zealand Journal of Zoology 28: 351-359.

21. Gill B, Whitaker T (1996) New Zealand frogs and reptiles. Auckland, New Zealand: David Bateman Ltd.,.

22. McCann C (1961) The introduced frogs of New Zealand. Tuatara 8: 107-120.

23. Medway L, Marshall AG (1975) Terrestrial vertebrates of the New Hebrides: origin and distribution. Philosophical transactions of the Royal Society of London Series B, Biological sciences 272: 423-465.

24. Moore JA (1961) The frogs of eastern New South Wales. Bulletin of the American Museum of Natural History 121: 151-385.

25. Neill WT (1964) Frogs introduced on islands. Quarterly Journal of the Florida Academy of Science 27: 127-130.

26. Pyke GH, White AW, Bishop PJ, Waldman B (2002) Habitat-use by the green and golden bell frog *Litoria aurea* in Australia and New Zealand. Australian Zoologist 32: 12-31.

27. Robb J (1973) Reptiles and amphibia. In: Williams GR, editor. The natural history of New Zealand: an ecological survey. Wellington, New Zealand: A. H. & A. W. Reed. pp. 285-303.

28. Robb J (1986) New Zealand amphibians and reptiles in colour. Auckland: Collins.

29. Thomas BW (1982) A review of the herpetofauna of southern New Zealand with some taxonomic considerations. Herpetofauna 14: 22-34.

30. Thomson GM (1922) The naturalisation of animals and plants in New Zealand. London: Cambridge University Press.

31. Thurley T, Bell BD (1994) Habitat distribution and predation on a western population of terrestrial *Leiopelma* (Anura: Leiopelmatidae) in the northern King country, New Zealand. New Zealand Journal of Zoology 21: 431-436.

32. West JA (1979) The occurence of some exotic reptiles and amphibians in New Zealand. Herpetofauna 10: 4-9.

33. Cree A (1984) Breeding biology, respiration and larval development of two introduced frogs (*Litoria raniformis* and *L. ewingi*). New Zealand Journal of Zoology 11: 179-188.

34. Cree A (1985) Water balance and nitrogen excretion of two introduced frogs (*Litoria raniformis* and *L. ewingi*). New Zealand Journal of Zoology 12: 341-348.

35. Gill BJ (1973) Distribution and habits of the brown tree frog *Litoria ewingi* Dumeril and Bibron in the Manawatu-Rangitikei region. Proceedings of the New Zealand Ecological Society 20: 31-34.

36. Low T (2003) The new nature. Camberwell, Australia: Penguin Books.

37. Marriner GR (1907) On the presence of another Australian frog in New Zealand. Transactions of the New Zealand Institute 39: 144-149.

38. Sharell R (1966) The tuatara, lizards, and frogs of New Zealand. London: Collins.

39. Eldredge LG (1994) Perspectives in aquatic exotic species management in the Pacific Islands: Vol I. Introductions of commercially significant aquatic organisms to the Pacific Islands. In: 78 SPREPSN, editor. Noumea, New Caledonia: South Pacific Commision.

40. Falanruw MC (1976) Life on Guam: savanna, old fields, roadsides. Guam: Guam Department of Education.

41. Hero JM, Gillespie GR, Lemckert F, Littlejohn MJ, Robertson P, et al. (2004) *Litoria raniformis* In: IUCN 2014. IUCN Red List of Threatened Species Version 3.1. http://www.iucnredlist.org. Accessed 18 March 2015.42. Hutton FW (1904) Index faunae novae zealandiae. London: Dulau & Co.

43. Tyler M (1982) The hylid frog genus *Litoria* Tschudi: an overview. Occasional Publications of the New Zealand Department of International Affairs Wildlife Service 2: 103-112.
