# Supplementary material for: Human-Assisted Invasions of Pacific Islands by Litoria Frogs: A Case Study of the Bleating Tree Frog on Lord Howe Island
Source: PLoS One. 2015 May 11;10(5):e0126287. doi: 10.1371/journal.pone.0126287 (PMC4427294; doi:10.1371/journal.pone.0126287)
Supplement: S2 Table — (DOC) [file pone.0126287.s002.doc]

**Supporting Information Table S2**: Collection localities on Lord Howe Island and in Eastern Australia of *Litoria dentata* samples.

| Species | Collection Locality |  |  | Sample Code | Tissue Code | GenBank Accession Number | |
| --- | --- | --- | --- | --- | --- | --- | --- |
|  |  | Longitude | Latitude |  |  | ND4 | Control Region |
| ***Litoria dentata*** |  |  |  |  |  |  |  |
| **Lord Howe Island** | LHIB Office grounds, LHI NSW | 31°31'44.6"S | 159°04'07.5"E | DEN001 | — | KM199718 | KM199696 |
|  | North Bay, LHI NSW | 31°30'59.5"S | 159°02'30.4"E | DEN002 | — | KM199718 | KM199696 |
|  | North Bay, LHI NSW | 31°30'59.5"S | 159°02'30.4"E | DEN003 | — | KM199718 | KM199696 |
|  | Old Settlement Beach, LHI NSW | 31°31'06.2"S | 159°03'13.9"E | DEN004 | — | KM199718 | KM199696 |
|  | Old Settlement Beach, LHI NSW | 31°31'06.2"S | 159°03'13.9"E | DEN005 | — | KM199718 | KM199696 |
|  | Old Settlement Beach, LHI NSW | 31°31'06.2"S | 159°03'13.9"E | DEN006 | — | KM199718 | KM199696 |
|  | Old Settlement Beach, LHI NSW | 31°31'06.2"S | 159°03'13.9"E | DEN007 | — | KM199718 | — |
|  | Old Settlement Beach, LHI NSW | 31°31'06.2"S | 159°03'13.9"E | DEN008 | — | KM199718 | — |
|  | Old Settlement Beach, LHI NSW | 31°31'06.2"S | 159°03'13.9"E | DEN009 | — | KM199718 | — |
|  | Old Settlement Beach, LHI NSW | 31°31'06.2"S | 159°03'13.9"E | DEN010 | — | KM199718 | — |
|  | Old Settlement Beach, LHI NSW | 31°31'06.2"S | 159°03'13.9"E | DEN012 | — | KM199718 | — |
|  | Moseley Ponds, LHI NSW | 31°32'30.2"S | 159°04'52.7"E | DEN021 | — | — | KM199696 |
|  | Moseley Ponds, LHI NSW | 31°32'30.2"S | 159°04'52.7"E | DEN022 | — | — | KM199696 |
|  | Moseley Ponds, LHI NSW | 31°32'30.2"S | 159°04'52.7"E | DEN023 | — | — | KM199696 |
|  | Moseley Ponds, LHI NSW | 31°32'30.2"S | 159°04'52.7"E | DEN024 | — | KM199718 | — |
|  | Moseley Ponds, LHI NSW | 31°32'30.2"S | 159°04'52.7"E | DEN025 | — | KM199718 | — |
|  | Moseley Ponds, LHI NSW | 31°32'30.2"S | 159°04'52.7"E | DEN026 | — | KM199718 | — |
|  | Moseley Ponds, LHI NSW | 31°32'30.2"S | 159°04'52.7"E | DEN027 | — | KM199718 | — |
|  | Moseley Ponds, LHI NSW | 31°32'30.2"S | 159°04'52.7"E | DEN028 | — | KM199718 | — |
|  | Moseley Ponds, LHI NSW | 31°32'30.2"S | 159°04'52.7"E | DEN029 | — | KM199718 | — |
|  | Moseley Ponds, LHI NSW | 31°32'30.2"S | 159°04'52.7"E | DEN030 | — | KM199718 | — |
|  | Moseley Ponds, LHI NSW | 31°32'30.2"S | 159°04'52.7"E | DEN031 | — | KM199718 | — |
|  | Moseley Ponds, LHI NSW | 31°32'30.2"S | 159°04'52.7"E | DEN032 | — | KM199718 | — |
|  | Moseley Ponds, LHI NSW | 31°32'30.2"S | 159°04'52.7"E | DEN034 | — | KM199718 | — |
|  | Moseley Ponds, LHI NSW | 31°32'30.2"S | 159°04'52.7"E | DEN035 | — | KM199718 | — |
|  | Moseley Ponds, LHI NSW | 31°32'30.2"S | 159°04'52.7"E | DEN036 | — | KM199718 | — |
|  | Moseley Ponds, LHI NSW | 31°32'30.2"S | 159°04'52.7"E | DEN037 | — | KM199718 | — |
|  | Moseley Ponds, LHI NSW | 31°32'30.2"S | 159°04'52.7"E | DEN038 | — | KM199718 | — |
|  | Moseley Ponds, LHI NSW | 31°32'30.2"S | 159°04'52.7"E | DEN039 | — | KM199718 | — |
|  | Moseley Ponds, LHI NSW | 31°32'30.2"S | 159°04'52.7"E | DEN040 | — | KM199718 | — |
|  | Moseley Ponds, LHI NSW | 31°32'30.2"S | 159°04'52.7"E | DEN041 | — | KM199718 | — |
|  | Moseley Ponds, LHI NSW | 31°32'30.2"S | 159°04'52.7"E | DEN042 | — | KM199718 | — |
|  | Moseley Ponds, LHI NSW | 31°32'30.2"S | 159°04'52.7"E | DEN043 | — | KM199718 | — |
|  | Moseley Ponds, LHI NSW | 31°32'30.2"S | 159°04'52.7"E | DEN044 | — | KM199718 | — |
|  | Goat House, LHI NSW | 31°33'31.0"S | 159°05'09.6"E | DEN120 | — | — | KM199696 |
|  | Soldiers Creek, LHI NSW | 31°33'07.7"S | 159°04'54.7"E | DEN121 | — | — | KM199696 |
| **Native Range** | Lighthouse Beach, Ballina NSW | 28°52'08.6"S | 153°35'25.3"E | DEN200 | — | KM199705 | KM199697 |
|  | Lighthouse Beach, Ballina NSW | 28°52'08.6"S | 153°35'25.3"E | DEN201 | — | KM199706 | — |
|  | Ballina Bowl, Ballina NSW | 28°51'19.4"S | 153°33'35.2"E | DEN202 | — | KM199705 | — |
|  | Ballina Bowl, Ballina NSW | 28°51'19.4"S | 153°33'35.2"E | DEN203 | — | KM199705 | — |
|  | Ballina Bowl, Ballina NSW | 28°51'19.4"S | 153°33'35.2"E | DEN204 | — | KM199707 | — |
|  | Ballina Bowl, Ballina NSW | 28°51'19.4"S | 153°33'35.2"E | DEN205 | — | KM199708 | KM199698 |
|  | Palmers Island, Yamba NSW | 29°25'12.4"S | 153°17'12.7"E | DEN206 | — | KM199709 | KM199699 |
|  | Palmers Island, Yamba NSW | 29°25'12.4"S | 153°17'12.7"E | DEN207 | — | KM199710 | KM199700 |
|  | Palmers Island, Yamba NSW | 29°25'12.4"S | 153°17'12.7"E | DEN208 | — | KM199710 | KM199700 |
|  | Palmers Island, Yamba NSW | 29°25'12.4"S | 153°17'12.7"E | DEN209 | — | KM199711 | KM199701 |
|  | Palmers Island, Yamba NSW | 29°25'12.4"S | 153°17'12.7"E | DEN210 | — | KM199712 | KM199699 |
|  | Palmers Island, Yamba NSW | 29°25'12.4"S | 153°17'12.7"E | DEN211 | — | KM199715 | KM199699 |
|  | McKittrick Park, Grafton NSW | 29°42'29.9"S | 152°56'10.3"E | DEN212 | — | KM199705 | KM199702 |
|  | McKittrick Park, Grafton NSW | 29°42'29.9"S | 152°56'10.3"E | DEN213 | — | KM199712 | KM199699 |
|  | McKittrick Park, Grafton NSW | 29°42'29.9"S | 152°56'10.3"E | DEN214 | — | KM199713 | KM199699 |
|  | McKittrick Park, Grafton NSW | 29°42'29.9"S | 152°56'10.3"E | DEN215 | — | KM199714 | — |
|  | McKittrick Park, Grafton NSW | 29°42'29.9"S | 152°56'10.3"E | DEN216 | — | KM199715 | — |
|  | McKittrick Park, Grafton NSW | 29°42'29.9"S | 152°56'10.3"E | DEN217 | — | KM199707 | KM199703 |
|  | Coffs Harbour Racecourse, Coffs Harbour NSW | 30°18'28.7"S | 153°07'36.4"E | DEN218 | — | KM199716 | — |
|  | Bananacoast Caravan Park, Coffs Harbour NSW | 30°16'07.0"S | 153°07'57.3"E | DEN219 | — | KM199712 | KM199699 |
|  | Bananacoast Caravan Park, Coffs Harbour NSW | 30°16'07.0"S | 153°07'57.3"E | DEN220 | — | KM199717 | KM199704 |
|  | Bananacoast Caravan Park, Coffs Harbour NSW | 30°16'07.0"S | 153°07'57.3"E | DEN221 | — | KM199717 | — |
|  | Dorroughby Education Centre, Tweed Valley, NSW | 28°39'29.9"S | 153°21'00.0"E | DEN230 | ABTC86376 | KM199705 | — |
|  | Glenreagh, NSW | 30°03'00.0"S | 152°58'59.9"E | DEN231 | ABTC86356 | KM199724 | — |
|  | Mann River Nature Reserve, NSW | — | — | DEN232 | ABCT25457 | KM199721 | — |
|  | Between Coutts Crossing and Glenreagh, N Coffs Harbour, NSW | 29°55'39.0"S | 152°55'01.9"E | DEN233 | ABTC86359 | KM199725 | — |
|  | 3.5k N Grafton on Casino Road, NSW | 29°39'59.0"S | 152°56'15.0"E | DEN234 | ABTC86207 | KM199705 | — |
|  | Brunswick Heads, NSW | 28°31'58.8"S | 153°33'00.0"E | DEN235 | ABTC86205 | KM199705 | — |
|  | Peacock Creek, Richmond Range, NSW | — | — | DEN236 | ABTC25752 | KM199722 | — |
|  | Whian Whian State Forest, NSW | — | — | DEN237 | ABTC25731 | KM199707 | — |
|  | Midginbil Hill, NSW | 28°28'58.8"S | 153°15'57.6"E | DEN238 | ABTC24826 | KM199705 | — |
|  | Ourimbah, NSW | 33°21'57.6"S | 151°21'57.6"E | DEN239 | ABTC1244 | KM199719 | — |
|  | Mernot State Forest, NSW | 31°41'29.0"S | 151°35'29.0"E | DEN240 | ABTC25254 | KM199719 | — |
|  | Mt Royal National Park, NSW | — | — | DEN241 | ABTC25236 | KM199720 | — |
|  | Homebush Bay, Sydney NSW | 17°45'00.0"S | 139°33'00.0"E | DEN242 | ABTC86208 | KM199723 | — |
| *Litoria electrica* | Burketown, QLD | 31°41'29.0"S | 151°35'29.0"E | — | ABTC16521 | KM199726 | — |
| *Litoria rubella* | Collaroy Homestead, NSW | 32°05'21.5"S | 150°04'52.7"E | — | ABTC01233 | KM199727 | — |
